# Perforated Meckel’s diverticulum in neonates: a report of six cases and systematic review of the literature

**DOI:** 10.1186/s43159-021-00154-z

**Published:** 2022-03-08

**Authors:** Naeem Liaqat, Anies Mahomed, Sajid Iqbal Nayyar, Nadeem Akhtar, Sajjad Ali, Naveed Haider

**Affiliations:** 1 Medicare Hospital, Rawalpindi, Pakistan; 2 Reconstructive Surgery, Nationwide Children’s, Columbus, OH USA; 3 King Faisal Specialist Hospital & Research Center, Jeddah, 21499 Saudi Arabia; 4 Children’s Hospital, Lahore, Pakistan; 5 Center for Colorectal and Pelvic Reconstruction, Nationwide Children’s, Ohio Columbus, USA; 6 PIMS Hospital, Islamabad, Pakistan; 7 Khyber Teaching Hospital, Peshawar, Pakistan; 8 Dera Ghazi Khan Medical College and DHQ Teaching Hospital, Dera Ghazi Khan, Pakistan

**Keywords:** Neonate, Meckel diverticulum, Neonatal, Pneumoperitoneum

## Abstract

**Background:**

Perforation of Meckel Diverticulum (MD) is a rare cause of pneumoperitoneum in neonates. We hereby report six cases of perforation of MD in neonates, with addition of 53 cases from systematic review of the literature. A systematic review was performed using Mesh terms “Neonate, Meckel Diverticulum, Perforation, Pneumoperitoneum.” All reports of perforated MD in the English literature were identified. Details of our 6 cases were analyzed in similar fashion.

**Results:**

A total of 3027 manuscripts were screened and 59 cases including 6 of our own were identified. The vast majority (78%) were female. Fifty patients (84.7%) presented in the newborn period. Half of the cases (52.5%) had associated anomalies and 13 neonates (22%) required oxygen supplementation including CPAP or ventilatory support before surgery. In 73% of the cases, a resection of gut was undertaken. Histopathological assessment in 44 cases (74.6%) revealed no ectopic gastric mucosa. Three cases demised prior to treatment. The outcome in the vast majority was excellent with 84.7% surviving and discharged well.

**Conclusion:**

Perforated MD is an unusual cause of a pneumoperitoneum in the newborns. Diagnosis is established at laparotomy and it rare to find ectopic mucosa histopathologically. The overall outcome is excellent.

## Introduction

Pneumoperitoneum is a serious condition in neonates requiring immediate surgical intervention. The most common causes in this age group are necrotizing enterocolitis and intestinal atresia including a host of idiopathic pathologies. A rare cause is a perforated Meckel diverticulum (MD), of which, only a limited number of cases have been reported to date [1–3].

Authors encountered few cases of perforated MD and it intrigued us to look into the literature. We had many unanswered questions, which we intended to answer. These include

1. As commonly said that MD is two times more common in males than females, does this rule also apply in neonates having perforated MD.
2. What may be the cause of perforation of MD.? Is it hypertrophied gastric mucosa?
3. What is the outcome in terms of survival of these neonates?
4. Do these neonates present late in neonatal life? If so, then some environmental factor may be involved and must be investigated.
5. Is there any particular pattern of presentations of perforated MD which may help us diagnose these patients early?
6. What may the risk factors for mortality in these patients?

In this systematic review, we intended to look for the presentation details and outcomes of the newborns with MD and tried to answer these questions. Here, we report six cases of perforated MD in neonates and their details are being summarized in Table 1.

**Table 1:** Details of all cases included in SR

Sr. no.		Year	Gestational age (weeks)	Gender	Age at presentation (days)	Weight (g)	Ectopic tissue (histopathology)	Management	Outcome	Associated anomalies	Pre-op ventilation
1	**Case 1**		37	M	3	3000	None	ETEA	Dis	None	No
2	**Case 2**		36	M	2	2800	None	ETEA	Dis	None	No
3	**Case 3**		37	M	2	2500	None	ETEA	Expired	Anorectal Malformation	No
4	**Case 4**		NR	M	5	2650	None	ETEA	Dis	None	No
5	**Case 5**		37	M	4	NR	None	Stoma formation	Dis	None	No
6	**Case 6**		37	M	3	NR	None	ETEA	Dis	Omphalocoele minor	No
7	Bindi [4]	2020	NR	M	3	NR	NR	ETEA	Dis	COVID-19	No
8	Charki [5]	2019	37	M	1	3500	None	ETEA	Dis	Omphalocoele minor	No
9	Wang [3]	2019	27	M	2	1370	None	ETEA	Dis	None	Yes
10	McKelvie [2]	2019	30	F	3	1120	None	ETEA	Dis	Cord prolapse	Yes
11	Orelaru [6]	2018	31	M	3	1900	None	ETEA	Dis	Product of IVF, twin pregnancy	Yes
12	Nhatrang [7]	2018	23	M	5	625	None	Ileostomy	Dis	None	Yes
13	Louati [8]	2017	37	M	NR	2350	None	ETEA	Dis	None	No
14	Jin [9]	2017	37	F	1	2350	None	ETEA	Dis	Omphalocoele, VSD, ASD	No
15	Ayari [10]	2016	28	M	1	1400	NR	ETEA	Dis	Chorioamnionitis	No
16	Ayari [10]	2016	26	M	7	750	NR	Ileostomy	Dis	Hyaline membrane disease	Yes
17	Masuko [1]	2016	34	M	2	1970	None	ETEA	Dis	None	Yes
18	Frooghi [11]	2016	37	M	3	3200	Gastric tissue	ETEA	Dis	None	No
19	Alvares [12]	2015	30	M	10	940	None	ETEA	Dis	Hyaline membrane disease	Yes
20	Borgi [13]	2014	29	M	1	1400	None	ETEA	Dis	Twin pregnancy, other twin dies	Yes
21	Smolkin [14]	2013	28	M	4	1200	None	ETEA	NR	PDA	Yes
22	Bertozzi [15]	2013	34	M	5	2500	None	Ileostomy	Dis	Mother—bilateral hydroureteronephrosis	No
23	Crankson [16]	2013	29	M	1	1640	None	ETEA	Dis	Abruptio placenta	No
24	Skelly [17]	2012	39	M	2	3280	None	ETEA	Dis	Infantile hypertrophic pyloric stenosis, skip segment HD	No
25	Qasim [18]	2012	37	M	7	2500	NR	ETEA	Death	None	No
26	Lee DS [19]	2012	37	M	1	2510	None	ETEA	Dis	None	No
27	Khan [20]	2012	29	F	6	650	None	ETEA	Dis	IGR, PDA, on inotropic support	Yes
28	Kampfen [21]	2011	37	F	18	4000	NR	ETEA	Dis	None	No
29	Anay [22]	2011	24	M	3	740	None	Ileostomy	Dis	Mother—PIH, DM (on Metmorphin)	Yes
30	Nakazawa [23]	2009	36	M	1	1776	None	Ileostomy	Dis	IUGR	No
31	Alkan [24]	2009	38	F	1	2800	None	ETEA	Dis	None	No
32	Aguayo [25]	2009	28	M	6	810	None	Ileostomy	Dis	Mother—PIH, HELLP syndrome, oligohydramnios, IUGR	No
33	Mavridis [26]	2008	37	M	1	3800	None	ETEA	Dis	Omphalocoele minor	No
34	Oyachi [27]	2007	37	M	17	3060	None	ETEA	Dis	None	No
35	Sy [28]	2006	40	F	3	3200	None	ETEA	Dis	Hirschsprung’s disease—mid transverse colon	No
36	Chang [29]	2006	33	M	2	2040	None	ETEA	Dis	None	No
37	Lim [30]	2005	39	M	9	3540	None	ETEA	Dis	None	No
38	Ojha [31]	2004	37	M	6	3000	None	ETEA	Dis	Segmental dilatation of ileum	No
39	Zahra [32]	2003	37	M	3	2070	None	NR	Dis	Mother—bronchial asthma, UTI	No
40	Okazaki [33]	2003	39	M	3	2698	None	NR	Dis	Mother—hyperthyroid (took methimazole). Child—needed anti-thyroid drugs for 10 days	No
41	Tekant [34]	2001	30	M	3	2600	Gastric mucosa	ETEA	Dis	Mother had high gastrin levels and positive H. Pylori	No
42	Kumar [35]	1998	NR	M	5	2300	None	ETEA	Dis	None	No
43	Gandy [36]	1997	37	M	8	4500	None	NR	Dis	Diabetic mother	No
44	Yeh [37]	1996	NR	M	8	NR	None	ETEA	Dis	None	No
45	Ford [38]	1992	37	NR	1	1900	Pancreatic tissue	Ileostomy	Death	VATER, vertebral anom, imperforate anus, tracheo-esoph fistula, absent right kidney, dysplastic left kid, single umbilical artery, oligohydramnios	Yes
46	Coppes [39]	1991	32	M	3	1780	None	ETEA	Dis	None	Yes
47	Khope [40]	1988	37	M	3	NR	Gastric Mucosa	ETEA	Dis	Undescended testis	No
48	Wright and Bhawandeen [41]	1986	37	M	1	3515	Both gastric and pancreatic	ETEA	Dis	Hydrocoele	No
49	Dalens [42]	1981	40	M	2	3650	Gastric	NR	Dis	None	No
50	Mcmanus [43]	1980	NR	M	1	2268	None	ETEA	Dis	None	No
51	Lin [44]	1978	36	M	4	2450	None	ETEA	Dis	None	No
52	De Oliveira [45]	1967	NR	NR	7	NR	NR	ETEA	Dis	None	No
53	Mestel [46]	1966	NR	NR	NR	NR	NR	NR	Dis	None	No
54	Mestel [46]	1966	NR	NR	NR	NR	NR	NR	Death	None	No
55	Roger [47]	1964	NR	M	1	2300	None	ETEA	Dis	None	No
56	Abramson [48]	1960	NR	F	5	3742	None	ETEA	Dis	None	No
57	Gilbert [49]	1958	34	M	1	2693	None	Autopsy	Death	Cord twisted around neck	No
58	Rosza and Gross [50]	1953	32	F	1	NR	None	Autopsy	Death	Meconeum ileus	No
59	Hunter [51]	1928	NR	M	4	NR	None	Died	Death	None	No

*M* male, *F* female, *ETEA* end to end anastomosis, *Dis* discharged, *NR* not reported, *VSD* ventricular septal defect, *ASD* atrial septal defect, *HD* Hirschsprung’s disease, *PDA* patent ductus arteriosus, *IUGR* intra-uterine growth retardation, *DM* diabetes mellitus

## Methodology

We conducted this systematic review with the aim to get all reports from the literature about MD perforation in neonates. We used PRISMA checklist to maintain the integrity and structure. It was performed using Mesh terms “Neonate, Meckel Diverticulum, Perforation, Pneumoperitoneum.” Three databases were accessed: PubMed, Google Scholar, and Cochrane. No filter for the time, language, or region was applied during the literature search, and all data to date (May 2021) was retrieved. We included all reports/studies reporting perforated MD in neonates. Two authors (NL and MA), acting independently identified full-text reports which were then collated. Included were all reports of perforation of MD, irrespective of the outcome. Further, reference lists of all those full texts were seen to identify any missing reports, and if found, it was included. Also, we went through the literature review table of these manuscripts to find any missing reports. Neonate was defined as any child within 30 days of life at the time of presentation. The following information was extracted from the reports; author, journal, year of publication, gestational age in weeks, gender, age at the time of presentation, the weight of the child at the time of presentation, associated anomaly, treatment strategy, histopathology report, in particular whether ectopic tissue was found, pre-operative history of ventilation, and outcome. Details of our own cases were similarly recorded on an Excel spreadsheet and analyzed. Along with simple descriptive statistics, we also conducted logistic regression analysis to look for the odd’s ratio (OR) for mortality among these factors in order to identify the risk factors.

## Results

A total of 3027 manuscripts were screened and 62 cases were identified. Nine cases were excluded as the manuscripts were published in languages other than English [52–60]. A total of 59 cases, including our own six cases, were finally included in the study (Table 1). The details of all the cases are summarized in Tables 1 and 2.

**Table 2:** Descriptive Statistics of reported cases in the literature

**Gestational age (weeks)**	
< 37 weeks	23 (39%)
≥ 37 weeks	25 (42.4%)
NR	11 (18.6%)
**Gender**	
Male	47 (79.7%)
Female	8 (13.6%)
NR	4 (6.8%)
**Age at presentation**	
**≤ 7 days**	50 (84.74%)
> 7 days	6 (10.16%)
NR	3 (5.1%)
**Weight**	
**< 2500 g**	25 (2.4%)
>+ 2500 g	24 (40.7%)
NR	10 (16.94%)
**Ectopic mucosa on histopathology**	
Gastric	4 (6.8%)
Pancreatic	1 (1.7%)
Both gastric and pancreatic	1 (1.7%)
No ectopic mucosa	44 (74.6%)
NR	9 (15.2%)
**Management**	
Resection and anastomosis	43 (72.9%)
Stoma formation	7 (11.9%)
NR	6 (10.2%)
Autopsy	3 (5.1%)
**Outcome**	
Death	7 (11.9%)
Discharge	51 (84.7%)
NR	1 (1.7%)
**Associated anomalies**	
Yes	31 (52.54%)
No	28 (47.45%)
**Pre-operative ventilation**	
Yes	13 (22%)
No	46 (78%)

Twenty-three patients (39%) were born prematurely, and the majority of patients (79.7%) were male. Fifty patients (84.7%) presented in the newborn period. Almost half of the cases (52.5%) reported other congenital anomalies. These anomalies included Omphalocele, anorectal malformation, Hirschsprung’s disease, meconium ileus, and many more (Table 1). Mothers of 11 neonates had some complications during gestation, including, bronchial asthma, UTI, Diabetes, PIH, HELLP syndrome, and abruptio placenta. Thirteen neonates (22%) required oxygen supplementation including CPAP or ventilatory support before surgery. Preoperative imaging rarely gives a clue as to the cause of the pneumoperitoneum. Only one case was suspected preoperatively and the rest diagnosed at laparotomy. In 73% of the cases, surgeons opted to resect the involved segment and restore the continuity of the gut. Histopathological assessment in 44 cases (74.6%) revealed no ectopic gastric mucosa. Three cases were diagnosed on autopsy as patients died before any treatment. The outcome in the vast majority was excellent with 84.7% of cases discharged well. Composite data are summarized in Table 2. Logistic regression showed that none of the factors were significantly associated with the mortality among these patients (Table 3).

**Table 3:** Logistic regression to look for factors leading to mortality

Factors	OR: 95%CI (range): ***P*** value
Pre-operative ventilation	0.556: (0.061–5.080): 0.603
Male gender	0.651: (0.063–6.708): 0.718
Presence of any ectopic mucosa	1.567: (0.156–15.768): 0.703
Presence of associated anomaly	1.235 :(0.251–7.071): 0.795
Low birth weight (weight less than 2500 g)	0.292 :(0.028–3.021): 0.302
Prematurity (gestational age less than 37 weeks)	0.698 :(0.106–4.607): 0.709

## Discussion

MD is a remnant of the omphalomesenteric duct, which normally regresses during the 5th–7th week of gestation. Its typically a 3–6-cm-long outpouching on the antimesenteric border, 50–75 cm from the ileo-caecal junction and usually contains all four intestine layers. In 30 to 50% of patients, it contains ectopic tissues which maybe gastric, pancreatic, colonic, duodenal, or endometrial. Despite being the most common congenital anomaly of the gastrointestinal tract, symptomatic manifestation in the neonatal period is rare. Complications may occur in up to 4% of cases, and in the symptomatic, intestinal perforation is seen fewer than 10% of cases [1]. Diverticular length and base diameter are well-known predisposing factors to complications with long, narrow-based diverticula thought to predispose to obstruction on the basis of intussusception and inflammation [3, 5, 7]. Common manifestations of neonatal MD include perforation, intussusception, segmental ileal dilatation, and ileal volvulus [8, 28, 61]. Bertozzi and colleagues [15] identified bowel obstruction (58.3%) and pneumoperitoneum (33.3%) as the most common clinical manifestations. Umbilical catheterization is a rare cause of iatrogenic perforation [61].

Typically, MD is synonymous with the rule of 2; seen in 2% of the population, twice as frequent in the male sex with two percent being symptomatic [15]. Our collective review of perforated cases found a significant male predominance with a ratio of 51 to 8. This trend is interesting and has not previously been identified and further study to explain this phenomenon is required.

The timing of presentation is also of interest as 84.7% of patients in this review presented within the first week of life. Some presented immediately after birth suggesting a peri or very early post-natal onset of pathology [23, 26, 45]. There is no evidence to suggest that the perforations occur antenatally and it would be rare for this to be detected as expectant mothers are not routinely subjected to ultrasound screening in the last days of pregnancy. Gilbert et al., reported a neonate who died before any intervention and suspected the perforation to be antenatal [49].

The etiology of perforated MD is elusive, and many theories have been put forth. In older children and adults, ulceration induced perforation secondary to gastric ectopic tissue within a MD is well recognized. Only 6.8% (*n* = 4) of cases in this study had documented gastric tissue within the MD suggesting that other factors are responsible [27]. Tekant et al. proposed H. Pylori infection as a possibility [34]. Some have postulated, but without much support, that this may be secondary to the separation of vitelline remnants from the abdominal wall [6]. Oyachi et al. proposed a knotting of a long MD around itself, leading to weakness in the intestine walls ultimately leading to perforation [27]. We however did not see evidence of this in our cases as the perforations were discreet and at the tip of the MD with no proximal obstruction.

A tenable hypothesis is diverticulitis within the pouch resulting in erosion of the wall with resultant perforation. In this review, although no ectopic mucosa was found (*n* = 44), inflammation was noted supporting the inflammatory hypothesis as a reasonable cause for the perforation. Although presentation is within the first week of life it is likely that the trigger for the inflammatory process occurs in the perinatal period with gradually progression. Notwithstanding this, a single case has been reported where abdominal distension with ventilatory support was required at birth and later surgery confirming a perforated MD [13].

Perforation of the appendix proximal to distal Hirschsprung’s disease is well documented. Skelly reported a case where the child had skip segment Hirschsprung’s disease, and a perforated MD [17].

The standard presentation for perforated MD is a clinically acute abdomen with X ray confirmation of free intra-abdominal air. Rarely, unusual manifestations such as a scrotal pneumatocele secondary a perforated MD are seen [39]. It is exceptional for a specific diagnosis to be made preoperatively and a definite diagnosis of MD is usually established on laparotomy. However, Ojha et al. reported a case of a neonate where a pre-operative abdominal X-ray showed a massively dilated gut loop with outpouching which raised the possibility of a perforated MD [31].

With respect to management, most surgeons, 72.9% (*n* = 43), opted for resection along with end-to-end anastomosis. However, in some cases, 10.2% (*n* = 6), due to the patient’s poor clinical status, surgeons opted for an ileostomy. More recently, laparoscopy has been utilized in the management of these cases [1]. In instances where patients are too unwell to be shifted to the operating room, exploration is performed in the NICU setting [14]. With adequate perioperative support, the outcome for these patients is excellent.

Perforated MD is a rare entity where the diagnosis is only made at exploration. The management involves a resection of the MD with primary or delayed anastomosis and the outcome is excellent. Ectopic gastric mucosa is not a frequent finding on histopathology and the pathogenesis of perforation is more likely to be related to an inflammatory process within the diverticulum.

In summary we found following answers:

1. Question: As commonly said that MD is two times more common in males than females, does this rule also apply in neonates having perforated MD?
  Answer: No, this rule does not apply in this cohort of patients. Male preponderance is much more (6.3:1)
2. Question: What may be the cause of perforation of MD. Is it gastric mucosa?
  Answer: Gastric mucosa is found in only 8.5% of cases. So, the cause remains largely unknown.
3. Question: What is the outcome in terms of survival of these neonates?
  Answer: Generally, these neonates have a good survival as other surgical conditions of this age group.
4. Question: Do these neonates present late in neonatal life? If so, then some environmental factor may be involved and must be investigated.
  Answer: Most of these patients present in early neonatal age, so we don’t presume the involvement of some environmental agents; however, the possibility can’t be ruled out.
5. Question: Is there any particular pattern of presentations of perforated MD which may help us diagnose these patients early?
  Answer: No, we did not find any particular pattern and generally it was non-specific presentation with intestinal obstruction.
6. What may the risk factors for mortality in these patients?
  Answer: We did not find any factor being significantly associated with the mortality.

## Data Availability

Available for review (if needed)

## Abbreviation

MD: Meckel’s diverticulum
